# Changes in Global Longitudinal Strain after TAVI: Additional Prognostic Value over Cardiac Damage in Patients with Severe Aortic Stenosis

**DOI:** 10.3390/jcm13133945

**Published:** 2024-07-05

**Authors:** Rinchyenkhand Myagmardorj, Federico Fortuni, Xavier Galloo, Takeru Nabeta, Maria Chiara Meucci, Steele C. Butcher, Frank van der Kley, Jeroen J. Bax, Nina Ajmone Marsan

**Affiliations:** 1 Department of Cardiology, Heart Lung Centre, Leiden University Medical Centre (LUMC), 2333 ZC Leiden, The Netherlands; fortuni.ff9@gmail.com (F.F.); x.galloo@lumc.nl (X.G.); t.nabeta@lumc.nl (T.N.); m.c.meucci@lumc.nl (M.C.M.); s.c.butcher@lumc.nl (S.C.B.); f.van_der_kley@lumc.nl (F.v.d.K.); j.j.bax@lumc.nl (J.J.B.); n.ajmone@lumc.nl (N.A.M.); 2 Department of Cardiology, San Giovanni Battista Hospital, 06034 Foligno, Italy; 3 Heart Center, University of Turku and Turku University Hospital, 20520 Turku, Finland

**Keywords:** left ventricular global longitudinal strain, cardiac damage staging, aortic stenosis, transcatheter aortic valve implantation

## Abstract

**Background:** Previous studies demonstrated the prognostic value of baseline cardiac damage staging as well as left ventricular global longitudinal strain (LVGLS) in patients undergoing transcatheter aortic valve implantation (TAVI). The aim of the present study was to evaluate the changes in cardiac damage stage and LVGLS after TAVI and to investigate their prognostic values when integrated into the follow-up assessment. **Methods:** Patients with severe aortic stenosis undergoing TAVI were hierarchically classified into cardiac damage stages based on echocardiographic criteria before TAVI and at a 6-month follow-up. At the same time, LVGLS was measured. The staging system included stage 0 = no signs of cardiac damage; stage 1 = LV damage; stage 2 = mitral or left atrial damage; stage 3 = pulmonary vasculature or tricuspid damage; and stage 4 = right ventricular damage. The primary endpoint was all-cause mortality. **Results:** A total of 620 patients were included. At follow-up, LVGLS significantly improved, and the improvement was similar among each baseline cardiac damage stage. Follow-up LVGLS values were divided into quintiles, and each quintile was integrated into the cardiac damage staging, leading to a reclassification of 308 (50%) patients. At the time of a median follow-up at 48 (IQR 31–71) months starting from the 6-month follow-up after TAVI, 262 (38%) patients had died. A multivariable Cox regression model showed that LVGLS-integrated cardiac damage staging at follow-up had an incremental prognostic value over the baseline assessment (HR per 1-stage increase 1.384; 95% CI 1.152–1.663; *p* < 0.001). **Conclusions:** The integration of LVGLS with conventional echocardiographic parameters of cardiac damage at a 6-month follow-up after TAVI can improve patient risk-stratification.

## 1. Introduction

Aortic stenosis (AS) is the most common type of valvular heart disease in the aging population of developed countries [1], and if left untreated, it is associated with progressive left ventricular (LV) damage and poor prognosis [2,3,4,5]. In particular, the increase in LV afterload related to severe AS causes LV hypertrophy and remodeling, with diastolic but ultimately also systolic dysfunction. The last one, along with the presence of symptoms, represents one of the major indications for intervention, and is therefore used to risk-stratify patients with severe AS [6,7]. Recently, a cardiac damage staging system based on conventional echocardiographic parameters has been proposed to depict potential abnormalities involving the whole heart in the presence of severe AS, therefore taking into account the left ventricle, the mitral valve, the left atrium, pulmonary circulation, the right ventricle (RV), and the tricuspid valve [8]. This staging system showed a strong prognostic value both when assessed before transcatheter aortic valve implantation (TAVI) and also during follow-up, when changes in the cardiac damage stage have occurred [9]. In addition, a more accurate assessment of LV function in AS patients has been demonstrated by using [10] LV global longitudinal strain (GLS), which has also been shown to improve the prognostic value of cardiac damage staging when integrated into the baseline assessment before TAVI [11]. However, there are still no data on the potential value of integrating LVGLS with the cardiac damage staging in the follow-up of patients undergoing TAVI, although both are expected to improve after the intervention [9,12,13,14,15]. Consequently, we aimed to evaluate the changes in cardiac damage staging integrated with LVGLS 6 months after TAVI and to investigate their association with long-term mortality.

## 2. Materials and Methods

### 2.1. Study Population

**Figure 1:**
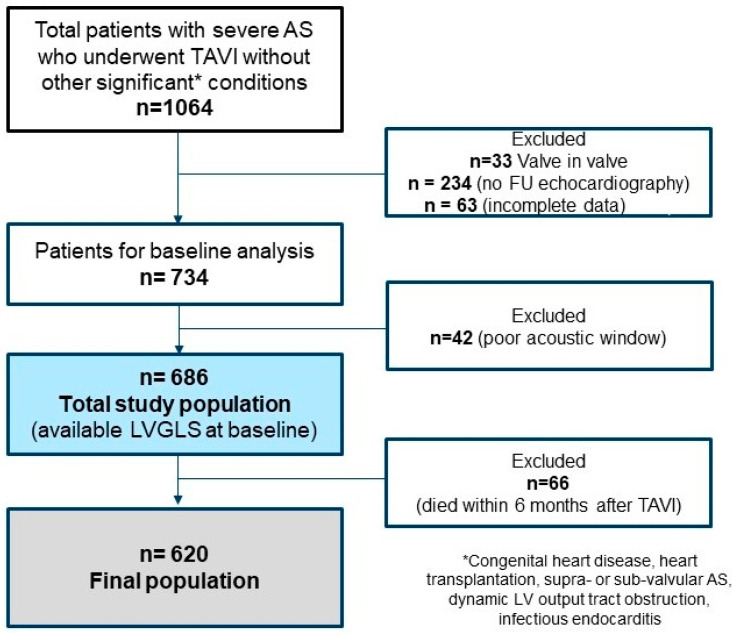
Study flow chart. From the total database of 1064 patients with severe AS who underwent TAVI, *n* = 620 patients were included in the final study population after the aforementioned exclusions. AS, aortic stenosis; FU, follow-up; LV, left ventricular; LVGLS, left ventricular global longitudinal strain; TAVI, transcatheter aortic valve implantation.

We retrospectively included patients with severe AS who underwent TAVI at the Leiden University Medical Center (Leiden, The Netherlands) from November 2007 to December 2019. Severe AS was diagnosed based on the following echocardiographic parameters: aortic valve area assessed with the continuity equation < 1.0 cm^2^ (or an indexed aortic valve area < 0.6 cm^2^/m^2^), mean aortic valve gradient ≥ 40 mmHg and/or peak aortic jet velocity ≥ 4 m/s [16]. The indication for TAVI, as well as the route of access and valve type, were decided by the local heart team. Patients with congenital heart disease, heart transplantation, supra- or sub-valvular AS, dynamic LV outflow obstruction, infectious endocarditis, and absence of analyzable baseline or follow-up echocardiography were excluded (Figure 1). Baseline demographic and clinical variables were evaluated pre-operatively. Clinical data included concomitant comorbidities, cardiovascular risk factors, EuroSCORE, NYHA functional class, medications, blood pressure, and the most recent serum hemoglobin and creatinine levels before TAVI.

### 2.2. Echocardiographic Assessment

All transthoracic echocardiographic (TTE) exams were performed by experienced sonographers using available ultrasound systems (Vivid 7 and E9 systems equipped with 3.5 MHz or M5S; General Electric Vingmed, Horten, Norway). TTE exams were performed at baseline and 6 months after TAVI. All strain measurements were performed by a single reader. The median time interval between the baseline echocardiography and TAVI was 1 day (interquartile range [IQR] 1–30 days). The median time interval between TAVI and the follow-up echocardiographic assessment was 6 (IQR 6–7) months.

Offline image data were digitally stored and retrospectively analyzed with dedicated software (EchoPAC versions 203 and 204, GE Medical Systems, Horten, Norway). From the parasternal long-axis view, linear dimensions of the left ventricle were measured, and the LV mass was calculated using Devereux’s formula [17]. The LV volumes and ejection fraction (EF) were traced and calculated using the biplane Simpson volumetric method, combining apical four- and two-chamber views [17]. From the apical 4-chamber view, peak early (E) and late (A) diastolic velocities were measured using pulsed-wave Doppler recordings of the transmitral flow [18]. Tissue Doppler imaging was used to measure e′ at both the lateral and septal sides of the mitral annulus, and measurements were averaged to derive the E/e′ ratio as an index of LV filling pressure [18]. The left atrial volume was measured using apical 4- and 2-chamber views, excluding the left atrial appendage and pulmonary veins [17]. The pulmonary artery systolic pressure was non-invasively estimated by adding the peak tricuspid regurgitation (TR) pressure gradient to the right atrial pressure, which was estimated based on the dimension and collapsibility during inspiration of the inferior vena cava, assessed from the subcostal view. The severities of the mitral and tricuspid regurgitation were evaluated using a multi-parametric approach based on current recommendations [19]. Tricuspid annular plane systolic excursion (TAPSE) was measured using the M-mode recordings of the lateral tricuspid annulus acquired from the apical 4-chamber view [17,20]. Continuous-wave Doppler recordings were acquired and used to measure the peak aortic jet velocity from apical 3- or 5-chamber views [16]. The mean and peak aortic transvalvular pressure gradients were calculated using the Bernoulli equation, and the aortic valve area was estimated using the continuity equation [16].

The apical 4-, 3-, and 2-chamber views were used to measure LVGLS at baseline and at the 6-month follow-up with dedicated software (EchoPAC versions 203 and 204, GE Medical Systems, Horten, Norway) [17]. The endocardial border was automatically traced, including the entire myocardium, and manually adjusted when necessary. LVGLS was automatically calculated as the average peak systolic strain of 17 LV segments and presented as an absolute value [16].

### 2.3. Cardiac Damage Staging

**Figure 2:**
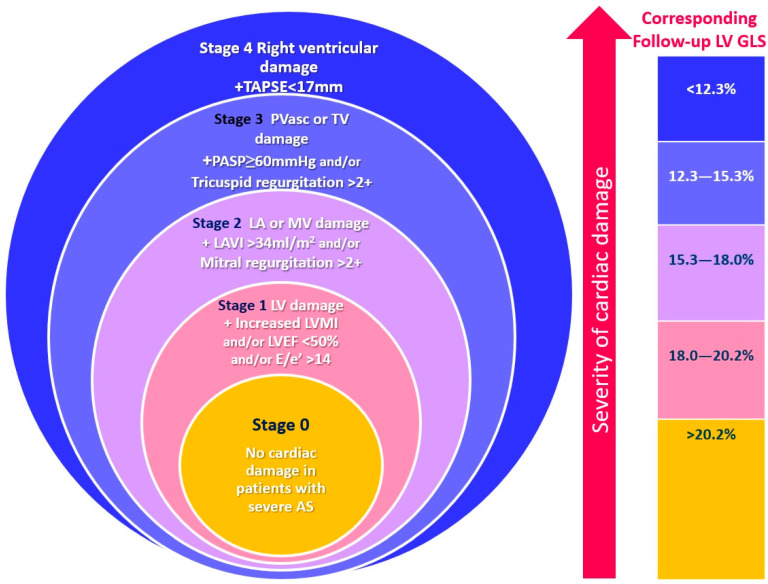
Stages of cardiac damage in severe aortic stenosis. The figure represents the echocardiography-based cardiac damage staging system that was applied before and after TAVI. AS, aortic stenosis; GLS, global longitudinal strain; LA, left atrial; LAVI, left atrial volume index; LV, left ventricular; LVEF, left ventricular ejection fraction, LVMI, left ventricular mass index; MV, mitral valve; PASP, pulmonary artery systolic pressure; PVasc, pulmonary vasculature; TAPSE, tricuspid annular plane systolic excursion; TR, tricuspid regurgitation; TV, tricuspid valve.

A validated echocardiography-based cardiac damage staging system was applied [8]. Patients were hierarchically classified into the cardiac damage stages (worst stages) if at least one of the proposed criteria was met (Figure 2). The staging included stage 0 = no signs of cardiac damage; stage 1 = LV damage, identified as LV ejection fraction < 50% and/or E/e′ > 14 and/or LV mass index > 115 g/m^2^ (male) or > 95 g/m^2^ (female); stage 2 = left atrial or mitral valve damage, identified as significant mitral regurgitation and/or left atrial volume index > 34 mL/m^2^; stage 3 = pulmonary vasculature or tricuspid valve damage, identified as pulmonary artery systolic pressure ≥ 60 mmHg and/or significant tricuspid regurgitation; stage 4 = RV damage, identified as TAPSE < 16 mm (Figure 2) [8]. Notably, AF was not included as a criterion to identify LA damage, as AF may be very common in patients undergoing TAVI and could lead to an overestimation of cardiac damage. Cardiac damage staging was performed at baseline and follow-up evaluation.

### 2.4. Follow-Up and Study Endpoint

We performed follow-ups of patients for incident all-cause mortality, which was the primary endpoint. The follow-up duration was censored at 7 years from the date of TAVI, when the baseline cardiac damage stage was considered. When assessing the association between the follow-up cardiac damage stage and primary endpoint, landmark analysis was performed in the timeframe from 6 months to 7 years after TAVI [21]. The endpoint data were collected from the departmental cardiology information system, which is linked to the municipal civil registries for survival assessment.

### 2.5. Statistical Analysis

Patient characteristics at baseline and follow-up were compared across cardiac damage stages (Figure 2). Categorical variables are presented as frequencies and percentages and were compared using the chi-square test. Continuous variables are shown as mean ± standard deviation if normally distributed and as median and IQR if non-normally distributed. Analysis of variance (ANOVA) was used to compare continuous variables with normal distribution, whereas Kruskal–Wallis’ test was applied to compare continuous variables with non-normal distribution. The Bonferroni correction was applied for multiple comparisons. Kaplan–Meier survival curves were obtained and analyzed, comparing the cumulative event rates across groups with log-rank test. A landmark analysis was performed to evaluate the association between the stage of cardiac damage at the 6-month follow-up and all-cause mortality at 7 years [21]. Univariable and multivariable Cox regression analyses were performed to identify the clinical and echocardiographic markers that could predict the occurrence of all-cause mortality during the follow-up. Clinical and echocardiographic variables were selected and included in the multivariable models based on statistical significance for the univariable analyses, avoiding collinearity. Specifically, to investigate the presence of collinearity between cardiac damage staging at the 6-month follow-up and LVGLS, we performed a correlation matrix of regression coefficients, which showed no significant correlation between these 2 parameters (regression coefficient 0.211), underlining a lack of collinearity that also justifies the integration of these variables into the novel classification of cardiac damage. Hazard ratios (HRs) and 95% confidence intervals (CIs) were calculated and reported. The intra-class correlation coefficients for intra- and inter-observer variability for LVGLS on 15 randomly selected patients were 0.962 and 0.895, demonstrating excellent agreement (Supplementary Table S1). A 2-sided *p* value < 0.05 was considered statistically significant. Statistical analysis was performed using SPSS software version 29.0 (SPSS Inc., Chicago, IL, USA).

## 3. Results

### 3.1. Baseline Clinical and Echocardiographic Characteristics

In Table 1 and Table 2, baseline clinical and echocardiographic characteristics for the overall population divided by cardiac damage stages are presented. At baseline, the proportions of patients allocated to each cardiac damage stage were 4% (*n* = 29), 11% (*n* = 73), 30% (*n* = 204), 31% (*n* = 217), and 24% (*n* = 163), corresponding to stages 0, 1, 2, 3, and 4. Overall, the mean age was 80 ± 7 years, 55% were men, 58% presented with NYHA classes III–IV HF symptoms, 19% of the patients had permanent or paroxysmal atrial fibrillation (AF), and the prevalence of AF increased with worsening stages of cardiac damage, together with EuroSCORE II and the use of loop diuretics (Table 1). Table 2 presents the baseline echocardiographic characteristics of the study population. Overall, the mean LV mass was increased, and individual indices of LV diastolic function, such as E/e′ and LA volume, were impaired. While the median LV ejection fraction was preserved, the mean LVGLS was reduced. With the worsening of cardiac damage, both LV linear and volumetric dimensions increased, and LV systolic function worsened, as did TAPSE.

### 3.2. Echocardiographic Follow-up after TAVI

**Figure 3:**
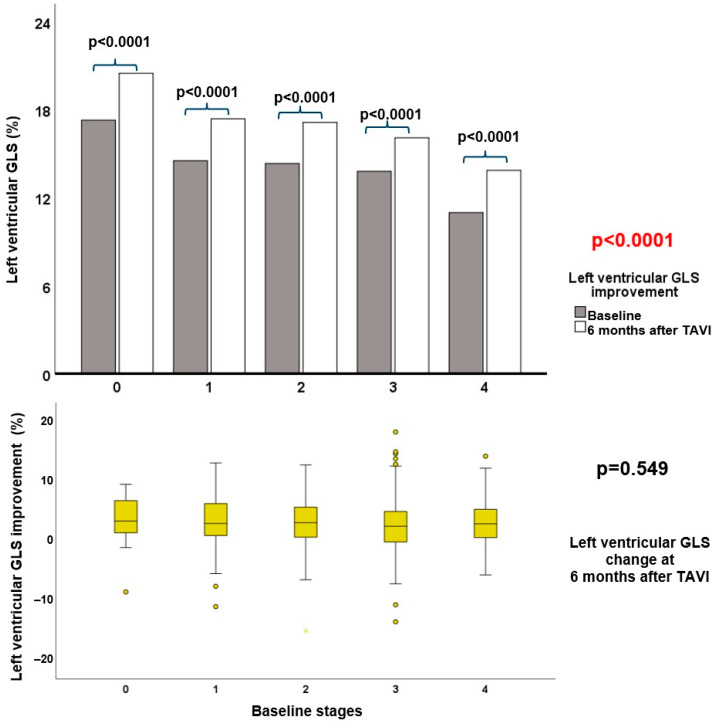
Changes in LVGLS over time by each cardiac damage stage. The upper bar chart illustrates the improvement in LVGLS from baseline to 6-month follow-up according to each baseline stage, while the lower box plot shows the extent of the change in LVGLS for each baseline cardiac damage stage (*n* = 620). A star represents extreme value and circles represents outliers. GLS, global longitudinal strain; LV, left ventricular; TAVI, transcatheter aortic valve implantation.

At the 6-month follow-up, 66 (10%) patients had died, and, therefore, 620 patients were included for paired analysis (Table 3). Compared to the baseline assessment, at the 6-month follow-up, there was a slight increase in LV dimensions accompanied by a decrease in LV mass. Moreover, both LV ejection fraction and LVGLS (13.6% versus 16.3%, *p* < 0.0001) improved as well as TAPSE in conjunction with significant decreases in MR and TR severity and pulmonary artery systolic pressures. At follow-up, 3% (*n* = 21) of the patients were classified as stage 0 (no signs of cardiac damage), 19% (*n* = 119) as stage 1 (LV damage), 49% (*n* = 304) as stage 2 (LV or mitral valve damage), 20% (*n* = 126) as stage 3 (pulmonary vasculature or tricuspid valve damage), and 9% (*n* = 50) as stage 4 (RV damage) (Supplementary Table S2). Remarkably, the improvement in LVGLS was similar (*p* = 0.565) among the baseline cardiac damage stages (Figure 3). To integrate LVGLS into the proposed staging system, follow-up LVGLS values were divided into quintiles and then assigned to stages of cardiac damage top down (>20.2% to stage 0; 18.0–20.2% stage 1; 15.3–18.0% stage 2; 12.3–15.3% stage 3; and <12.3% stage 4) as an additional criterion to identify the specific cardiac stage as shown in Figure 2, leading to a reclassification of 308 (50%) patients.

### 3.3. Association between Follow-up LVGLS-Integrated Cardiac Damage Staging and Long-Term All-Cause Mortality

**Figure 4:**
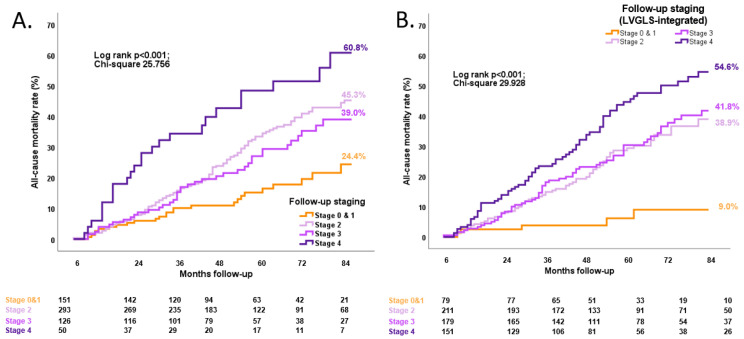
Patients’ event rates according to the follow-up cardiac damage stage. Kaplan–Meier survival curves at 7-year follow-up for all-cause deaths in patients stratified according to follow-up conventional cardiac damage stage (**A**), and according to LVGLS-integrated cardiac damage stage at follow-up (**B**) after transcatheter aortic valve implantation (*n* = 620). Due to the comparably lower number of patients at stage 0, patients at stages 0 and 1 are combined as one group. LVGLS, left ventricular global longitudinal strain.

**Figure 5:**
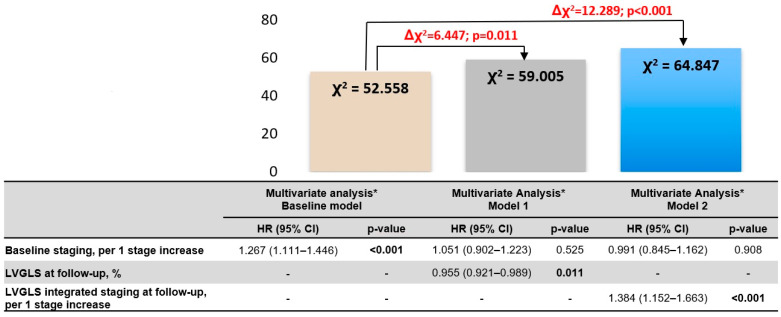
Incremental prognostic values adjusted association between follow-up cardiac damage staging and all-cause mortality after transcatheter aortic valve implantation. The figure shows the incremental prognostic value of the LVGLS assessment as well as the LVGLS-integrated cardiac damage staging system at 6 months after TAVI over cardiac damage before TAVI. CI, confidence interval; HR, hazard ratio; LVGLS, left ventricular global longitudinal strain. Bold values represent significant *p* values (<0.05). * Adjusted for male gender, coronary artery disease, use of diuretics, previous cardiac surgery, peripheral artery disease, smoking, mean aortic valve gradient (mmHg), hemoglobin (g/dL), creatinine (mg/dL).

At the time of a median follow-up of 48 months (IQR 31–71 months) starting from the 6-month follow-up after TAVI, 262 (38%) patients had died. Due to the comparably lower number of patients assigned to stages 0 and 1, they were merged and assigned to stage 1 in the survival analysis. Kaplan–Meier (KM) curves showed that survival rates in patients stratified by both the original follow-up staging as well as the new LVGLS-integrated cardiac damage staging were significantly different (overall log rank *p* < 0.0001, for each KM survival analysis) (Figure 4). Multivariable Cox regression models were built based on the results of the univariable Cox regression analysis (Table 4). Notably, after correcting for potential confounders, including EuroSCORE II and baseline assessment of cardiac damage before TAVI, both LVGLS at the 6-month follow-up (HR 0.955, CI 0.921–0.989, *p* = 0.011) and the LVGLS-integrated cardiac damage staging at the 6-month follow-up (HR 1.384, CI 1.152–1.663, *p* < 0.001) showed an independent association with the primary endpoint (Table 4). Moreover, when the LVGLS at the 6-month follow-up as well as the LVGLS-integrated cardiac damage staging assessed at follow-up were added to a basal model separately (Models 1 and 2), including cardiac damage staging before TAVI, results showed significant incremental prognostic values, underlining the importance of re-assessing cardiac damage at a follow-up after TAVI and integrating it with LVGLS assessment (Figure 5). In addition, when LVGLS assessed at the 6-month follow-up was added to a basal model including conventional cardiac damage staging at the 6-month follow-up, this showed an incremental prognostic value, which also formally justifies the integration of these parameters (LVGLS and cardiac damage staging at 6-month follow-up) to improve risk stratification (Supplementary Figure S1).

**Table 4:** Univariate and multivariate Cox regression analysis for all causes of death (*n* = 620).

	Univariate Analysis		Multivariate Analysis * Model 1	Multivariate Analysis * Model 2		
	HR (95% CI)	*p*-Value	HR (95% CI)	*p*-Value	HR (95% CI)	*p*-Value
Age, years	0.987 (0.969–1.006)	0.174				
Male gender	1.474 (1.105–1.966)	**0.008**	1.258 (0.896–1.765)	0.186	1.276 (0.912–1.786)	0.155
Body mass index (kg/m^2^)	0.998 (0.966–1.031)	0.909				
Coronary artery disease	1.481 (1.101–1.991)	**0.009**	1.078 (0.760–1.530)	0.672	1.087 (0.766–1.543)	0.640
Previous myocardial infarction	1.420 (1.036–1.946)	**0.029**				
Diuretic usage at baseline	1.579 (1.170–2.131)	**0.003**	1.362 (0.995–1.864)	0.054	1.322 (0.965–1.812)	0.082
Atrial fibrillation	1.277 (0.913–1.787)	0.153				
Previous cardiac surgery	1.738 (1.283–2.355)	**0.001**	1.254 (0.846–1.859)	0.260	1.211 (0.818–1.791)	0.339
Diabetes mellitus	1.248 (0.921–1.692)	0.153				
Hypertension	0.985 (0.714–1.358)	0.925				
Peripheral artery disease	1.679 (1.258–2.241)	**<0.001**	1.358 (0.989–1.865)	0.058	1.365 (0.994–1.874)	0.054
Smoking	1.390 (1.004–1.926)	**0.048**	1.329 (0.941–1.877)	0.106	1.361 (0.963–1.923)	0.081
Chronic obstructive pulmonary disease	1.306 (0.927–1.840)	0.126				
NYHA III–IV	1.137 (0.849–1.522)	0.389				
AV area, indexed	0.879 (0.366–2.113)	0.773				
AV mean gradient, mmHg	0.991 (0.982–0.999)	**0.036**	1.002 (0.992–1.011)	0.760	1.002 (0.992–1.012)	0.701
EuroSCORE II	1.060 (1.032–1.088)	**<0.0001**	-	-	-	-
Hemoglobin (g/dL)	0.895 (0.828–0.968)	**0.005**	0.857 (0.784–0.936)	**<0.001**	0.857 (0.785–0.934)	**<0.001**
Creatinine (mg/dL)	1.219 (1.088–1.366)	**<0.001**	1.165 (1.024–1.326)	**0.021**	1.164 (1.025–1.322)	**0.020**
Baseline staging, per 1 stage increase	1.147 (1.002–1.312)	**0.047**	1.051 (0.902–1.223)	0.525	0.991 (0.845–1.162)	0.908
Baseline LVGLS (%)	0.973 (0.942–1.006)	0.105				
LVGLS at follow-up	0.940 (0.913–0.968)	**<0.001**	0.955 (0.921–0.989)	**0.011**	-	-
Follow-up LVGLS-integrated staging, per 1 stage increase	1.355 (1.161–1.582)	**<0.001**	-	-	1.384 (1.152–1.663)	**<0.001**

AV, aortic valve; CI, Confidence interval; EuroSCORE, European System for Cardiac Operative Risk Evaluation; HR, hazard ratio; LVGLS, left ventricular global longitudinal strain; NYHA, New York Heart Association. Bold values represent significant *p* values (<0.05). * Landmark analysis was performed for multivariate Cox regression analysis.

## 4. Discussion

The current study showed that both the cardiac damage stage and LVGLS significantly improved after TAVI, and the improvement of LVGLS was similar across the different baseline cardiac damage stages. Furthermore, both the novel LVGLS-integrated cardiac damage staging system assessed at the 6-month follow-up after TAVI and the follow-up LVGLS demonstrated incremental prognostic value over conventional baseline cardiac damage staging and baseline LVGLS assessment. These findings underline the importance of following up on patients after TAVI and re-assessing them using advanced echocardiography to improve their risk stratification.

### 4.1. The Improvement of Cardiac Damage and LVGLS after TAVI

Severe AS causes significant LV remodeling, which consists mainly of LV hypertrophy as functional compensation for the increased LV afterload [22,23,24]. However, LV hypertrophy is often accompanied by myocardial damage and fibrosis, which may become irreversible and lead to LV dysfunction [25,26]. Current guidelines on valvular heart disease recommend considering AS-related symptoms and LVEF impairment to identify the optimal timing for intervention in patients with severe AS [16,27]. Nevertheless, when LV damage due to AS-related remodeling occurs, the LVEF can still be preserved. On the contrary, LVGLS showed a strong correlation with myocardial fibrosis and may be a superior indicator for the assessment of LV damage as compared to sole use of the LVEF [26,28,29,30]. In addition, damage at a different cardiac level than the left ventricle, as previously proposed by applying the staging system, further helped in characterizing and risk-stratifying these patients [8,9,11].

Although previous studies showed an improvement in the LVEF and GLS after AVR, no study so far assessed the difference in response of LVGLS after TAVI according to different cardiac damage stages and, therefore, different pre-TAVI remodeling grades. Former studies [13,14,15] showed that LV systolic function significantly improved in terms of both LVEF and GLS after TAVI. They highlighted that functional LV improvement was more pronounced in patients with impaired LV systolic function compared to those with more preserved indices of LV systolic function. Similarly, Sato et al. showed that patients with both normal and reduced pre-TAVI LVEF had similar GLS improvement 6 months after TAVI [31]. The current study further confirms the beneficial effect of TAVI on LVGLS independently of the grade of pre-TAVI AS-related remodeling since all patients showed an improvement in LVGLS regardless of the pre-TAVI stage of cardiac damage. This may have important implications for clinical practice since patients with advanced cardiac damage could also benefit from TAVI and experience a significant and relatively rapid improvement in LVGLS and, therefore, in cardiac function.

### 4.2. The Prognostic Value of Follow-Up Cardiac Damage Staging and LVGLS

Despite the abundant evidence on the importance of staging cardiac damage in patients with severe AS undergoing AV intervention [32] and on the use of LVGLS for the risk stratification of patients with severe AS, there are only a few studies that attempted to incorporate baseline LVGLS into the cardiac damage staging system [11,33]. Vollema et al. divided pre-TAVI LVGLS into quintiles (>18%, 15.8–18%, 13.2–15.8%, 10–13.2%, and <10%) and allocated them to cardiac damage stages from 0 to 4, leading to a reclassification of cardiac damage in a significant number of patients compared to the sole use of conventional echocardiographic parameters [11].

Moreover, the integration of LVGLS into the originally proposed staging of cardiac damage showed an incremental prognostic value over clinical characteristics and conventional stages of cardiac damage [11]. In the present study, LVGLS assessed at the 6-month follow-up after TAVI was considered, and, therefore, the LVGLS quintiles were significantly higher compared to the previous study (i.e., >20.2%, 18–20.2%, 15.3–18%, 12.3–15.3%, and <12.3%), and the integration of LVGLS into cardiac damage stages led to the reclassification of 308 patients (almost 50% of the total cohort). Notably, both LVGLS assessed 6 months after TAVI and the integration of LVGLS with follow-up cardiac damage stages resulted in incremental prognostic value over the baseline assessment. Our results, therefore, corroborate previous findings [11] on the importance of integrating advanced echocardiography to assess cardiac damage and, differently from the study by Vollema et al. [11] that focused only on pre-TAVI assessment, extend this concept to the follow-up of patients after TAVI. This study, therefore, suggests the importance of the follow-up evaluation after TAVI, when risk stratification should be repeated considering the changes that have occurred in response to the intervention to identify those patients still at high risk who might require closer monitoring. As can be noted from Figure 4, both conventional cardiac damage staging and LVGLS-integrated cardiac damage staging at follow-up were not able to demonstrate any significant difference in prognosis between stages 3 and 2. However, both classifications showed a worse prognosis for stage 4 and better outcome assessments for stages 0 or 1. Moreover, when LVGLS was integrated with the conventional cardiac damage staging, this novel classification performed better in identifying the better prognosis of patients in stages 0 or 1 at a 6-month follow-up after TAVI, underlining its additional value compared to the sole use of conventional echocardiographic parameters. Future research may focus on the use of artificial intelligence (AI) to identify cardiac damage stages based on echocardiographic images, show how these change after AVR, and maybe provide AI-based automatic tools to refine the risk stratification of patients with severe AS undergoing TAVI.

### 4.3. Limitations

The present study is limited by its retrospective and single-center design. This may have introduced selection and referral biases as patients underwent TAVI based on the decision of the heart team as recommended by contemporary guidelines. Although LVGLS was measured by using vendor-dependent software (EchoPAC versions 203 and 204, GE Medical Systems, Horten, Norway), recent studies showed only limited differences between vendors in the assessment of LVGLS [34]. Moreover, the current study did not assess the integration of segmental LV longitudinal strain and peak atrial longitudinal strain into the conventional cardiac damage classification, which may be further investigated in future studies.

## 5. Conclusions

LVGLS improves at follow-up after TAVI independently of the cardiac damage stage at baseline. The integration of LVGLS with conventional echocardiographic parameters of cardiac damage at 6-month follow-up after TAVI is of additional prognostic value compared to the baseline assessment.

## Figures and Tables

**Table 1:** Baseline characteristics according to cardiac damage staging at baseline.

	Total Population (*n* = 686)	Baseline					*p*-Value
		Stage 0 (*n* = 29)	Stage 1 (*n* = 73)	Stage 2 (*n* = 204)	Stage 3 (*n* = 217)	Stage 4 (*n* = 163)	
Age (years)	79.9 ± 7.3	78.8 ± 7.7	78.4 ± 8.5	80.3 ± 5.6	80.9 ± 7.2	79.1 ± 8.6	**0.035**
Male gender, *n* (%)	375 (55)	16 (55)	47 (64)	111 (54)	103 (48)	98 (60)	0.054
Body mass index (kg/m^2^)	26.5 ± 4.4	26.2 ± 3.9	26.9 ± 5.9	27.3 ± 4.3	25.7 ± 4.3 ^§^	26.3 ± 3.7	**0.007**
Body surface area (m^2^)	1.9 ± 0.2	1.9 ± 0.2	1.9 ± 0.2	1.9 ± 0.2	1.8 ± 0.2 ^§^	1.9 ± 0.2	0.050
Hypertension, *n* (%)	514 (76)	19 (68)	58 (80)	155 (78)	161 (76)	121 (75)	0.725
Diabetes mellitus, *n* (%)	196 (29)	6 (21)	23 (32)	68 (34)	47 (22)	52 (32)	0.057
Atrial fibrillation, *n* (%)	129 (19)	1 (3)	3 (4)	24 (12)	42 (19)	59 (36)	**<0.0001**
Pacemaker, *n* (%)	85 (12)	2 (7)	5 (7)	19 (9)	31 (14)	28 (17)	0.065
Hyperlipidemia, *n* (%)	436 (65)	18 (64)	40 (55)	129 (65)	143 (67)	106 (65)	0.446
Coronary artery disease, *n* (%)	406 (60)	14 (50)	42 (58)	115 (57)	122 (57)	113 (69)	0.066
Previous cardiac surgery, *n* (%)	135 (20)	3 (10)	9 (12)	35 (17)	29 (13)	59 (36)	**<0.0001**
Previous myocardial infarction, *n* (%)	153 (23)	4 (14)	18 (25)	36 (18)	46 (21)	49 (30)	0.056
Smoking, *n* (%)	139 (21)	5 (17)	22 (30)	44 (23)	32 (15)	36 (23)	0.078
Chronic obstructive pulmonary disease, *n* (%)	141 (22)	9 (31)	19 (26)	45 (23)	31 (15)	37 (24)	0.104
Peripheral artery disease, *n* (%)	192 (28)	9 (32)	23 (32)	56 (28)	51 (24)	53 (33)	0.390
EuroSCORE II (%)	3.2 (2.0–5.4)	2.0 (1.6–3.5)	2.7 (1.7–4.4)	3.0 (1.9–4.7)	3.0 (2.1–4.6)	4.7 (2.8–8.5) ^†‡§‖^	**<0.0001**
NYHA class III or IV, *n* (%)	389 (58)	13 (48)	38 (54)	106 (54)	128 (60)	104 (65)	0.129
Hemoglobin (g/dL)	12.5 ± 1.7	13.0 ± 1.6	12.7 ± 1.6	12.4 ± 1.9	12.4 ± 1.7	12.7 ± 1.6	0.118
Creatinine (mg/dL)	1.2 ± 0.8	1.1 ± 0.3	1.3 ± 1.4	1.2 ± 0.5	1.1 ± 0.9	1.3 ± 0.7	0.141
Medication Beta-blocker, *n* (%)	406 (61)	16 (57)	43 (59)	112 (56)	125 (60)	110 (69)	0.175
ACEi/ARB, *n* (%)	369 (55)	17 (61)	37 (51)	112 (56)	108 (51)	95 (59)	0.498
Calcium antagonist, *n* (%)	175 (26)	5 (18)	18 (25)	56 (28)	45 (21)	51 (32)	0.157
Diuretics, *n* (%)	378 (56)	13 (46.4)	31 (43)	103 (52)	123 (59)	108 (68)	**0.002**
Aspirin, *n* (%)	311 (47)	18 (67)	40 (56)	104 (53)	91 (44)	58 (37)	**0.002**
OAC/NOAC, *n* (%)	258 (39)	3 (11)	19 (27)	51 (26)	92 (44)	93 (59)	**<0.0001**
Statin, *n* (%)	441 (66)	20 (71)	43 (59)	138 (69)	135 (64)	105 (66)	0.514

Continuous variables are presented as mean ± SD or median [interquartile range]. Categorical variables are expressed as absolute numbers (%). The boldface values indicate statistical significance. ACEi, angiotensin-converting enzyme inhibitor; ARB, angiotensin II receptor blocker; EuroSCORE, European System for Cardiac Operative Risk Evaluation; NOAC, non-vitamin K oral anticoagulant; NYHA, New York Heart Association; OAC, oral anticoagulant. *p*-Values depict differences between stages of cardiac damage and are calculated by ANOVA and Kruskal–Wallis H test for continuous data (with normal and non-normal distribution, respectively), and by chi-square test for categorical data. ^†^ *p* Value < 0.05 vs. stage 0 with Bonferonni post hoc analysis. ^‡^ *p* Value < 0.05 vs. stage 1 with Bonferroni post hoc analysis. ^§^ *p* Value < 0.05 vs. stage 2 with Bonferonni post hoc analysis. ^‖^ *p* Value < 0.05 vs. stage 3 with Bonferonni post hoc analysis.

**Table 2:** Baseline echocardiographic characteristics according to cardiac damage staging at baseline.

	Total Population (*n* = 686)	Baseline					*p*-Value
		Stage 0 (*n* = 29)	Stage 1 (*n* = 73)	Stage 2 (*n* = 204)	Stage 3 (*n* = 217)	Stage 4 (*n* = 163)	
LV end-diastolic diameter indexed (mm/m^2^)	25.3 ± 4.5	22.2 ± 3.6	25.6 ± 3.9 ^†^	25.1 ± 4.0 ^†^	25.3 ± 4.5 ^†^	26.0 ± 5.1 ^†^	**0.001**
LV end-diastolic volume (mL/m^2^)	47.7 (37.6–64.0)	37.0 (30.0–45.7)	45.5 (35.8–56.9)	47.8 (37.9–61.6) ^†^	48.7 (38.6–64.0) ^†^	52.2 (38.1–74.2) ^†^	**<0.0001**
LV mass index (g/m^2^)	126.4 ± 38.4	84.6 ± 17.2	122.7 ± 35.1 ^†^	130.6 ± 38.6 ^†^	127.4 ± 39.2 ^†^	129.1 ± 37.0 ^†^	**<0.0001**
LV ejection fraction (%)	58.0 (46.3–65.0)	65.0 (59.5–71.0)	59.6 (50.7–66.1)	60.0 (52.0–66.0) ^†^	58.0 (47.3–65.0) ^†^	48.0 (36.9–60.0) ^†‡§‖^	**<0.0001**
LV global longitudinal strain (%)	13.4 ± 4.2	17.2 ± 3.4	14.5 ± 3.4	14.3 ± 3.7	13.7 ± 4.1	10.7 ± 4.1	**<0.0001**
E/e′ ratio	16.7 (12.0–24.3)	10.9 (8.6–12.0)	15.8 (11.1–21.0)	16.9 (12.5–25.3) ^†^	16.8 (12.5–25.0) ^†^	18.1 (12.9–25.7) ^†^	**<0.0001**
Left atrial volume index (mL/m^2^)	44.6 ± 16.4	24.4 ± 4.4	27.8 ± 4.4	46.8 ± 11.3 ^†‡^	47.7 ± 18.5 ^†‡^	49.0 ± 16.4 ^†‡^	**<0.0001**
Significant mitral regurgitation, *n* (%)	149 (22)	-	-	34 (17)	65 (31)	50 (31)	**<0.0001**
Systolic pulmonary arterial pressure (mmHg)	34.5 ± 15.1	26.8 ± 11.4	26.7 ± 13.5	29.2 ± 13.4	40.9 ± 13.5 ^†‡§^	37.6 ± 15.9 ^†‡§^	**<0.0001**
Significant tricuspid regurgitation, *n* (%)	309 (45)	-	-	-	210 (97)	99 (61)	**<0.0001**
Tricuspid annular plane systolic excursion (mm)	18.8 ± 4.5	19.6 ± 2.5	20.7 ± 4.1	21.1 ± 3.7	20.1 ± 3.0 ^§^	13.0 ± 2.0 ^†‡§‖^	**<0.0001**
Mean aortic valve gradient (mmHg)	41.1 ± 17.3	45.7 ± 13.6	43.3 ± 18.2	45.3 ± 18.0	41.0 ± 17.0	34.3 ± 15.1 ^†‡§‖^	**<0.0001**
Peak aortic jet velocity (m/s)	3.9 ± 0.8	4.2 ± 0.6	4.0 ± 0.8	4.1 ± 0.8	3.9 ± 0.8 ^§^	3.6 ± 0.8 ^†‡§‖^	**<0.0001**
Indexed aortic valve area (cm^2^/m^2^)	0.4 ± 0.2	0.4 ± 0.1	0.5 ± 0.2	0.4 ± 0.2	0.5 ± 0.2	0.4 ± 0.2	0.520

Continuous variables are presented as mean ± SD or median [interquartile range]. Categorical variables are expressed as absolute number (%). The boldface values indicate statistical significance. LV, left ventricular. *p*-Values depict differences between stages of cardiac damage and are calculated by ANOVA and Kruskal–Wallis H test for continuous data (with normal and non-normal distribution, respectively), and by chi-square test for categorical data. ^†^ *p* Value < 0.05 vs. stage 0 with Bonferonni post hoc analysis. ^‡^ *p* Value < 0.05 vs. stage 1 with Bonferroni post hoc analysis. ^§^ *p* Value < 0.05 vs. stage 2 with Bonferonni post hoc analysis. ^‖^ *p* Value < 0.05 vs. stage 3 with Bonferonni post hoc analysis.

**Table 3:** Comparison between baseline and 6-month follow-up echocardiographic characteristics (*n* = 620).

	Baseline (*n* = 620)	Follow-Up (*n* = 620)	*p*-Value
LV end-diastolic diameter indexed (mm/m^2^)	25.2 ± 4.5	26.2 ± 3.9	**<0.0001**
LV end-diastolic volume (mL/m^2^)	47.7 (37.6–64.0)	45.9 (38.5–56.4)	**<0.0001**
LV mass index (g/m^2^)	125.1 ± 38.5	118.2 ± 29.9	**<0.0001**
LV global longitudinal strain (%)	13.6 ± 4.2	16.3 ± 4.6	**<0.0001**
LV ejection fraction (%)	58.0 (46.1–65.0)	61.5 (53.3–69.3)	**<0.0001**
E/e′ ratio	16.7 (12.0–24.3)	18.8 (14.2–27.0)	**<0.0001**
Left atrial volume index (mL/m^2^)	44.3 ± 16.5	43.4 ± 16.8	0.107
Significant mitral regurgitation, *n* (%)	126 (21)	80 (13)	**<0.0001**
Systolic pulmonary arterial pressure (mmHg)	34.4 ± 14.8	31.0 ± 13.6	**<0.0001**
Significant tricuspid regurgitation, *n* (%)	274 (44)	140 (23)	**<0.0001**
Tricuspid annular plane systolic excursion (mm)	19.0 ± 4.5	20.0 ± 3.1	**<0.0001**
Mean aortic valve gradient (mmHg)	41.8 ± 17.2	9.8 ± 4.2	**<0.0001**
Peak aortic jet velocity (m/s)	4.0 ± 0.8	2.1 ± 0.4	**<0.0001**

Continuous variables are presented as mean ± SD or median [interquartile range]. Categorical variables are expressed as absolute number (%). The boldface values indicate statistical significance. LV, left ventricular. *p*-Values depict differences between stages of cardiac damage and are calculated by ANOVA and Kruskal–Wallis H test for continuous data (with normal and non-normal distribution, respectively), and by chi-square test for categorical data.

## Data Availability

The data supporting the findings of the study are available from the corresponding author, upon reasonable request.

## Supplementary Materials

The following supporting information can be downloaded at: https://www.mdpi.com/article/10.3390/jcm13133945/s1, Figure S1: Incremental prognostic value of follow-up LVGLS over conventional cardiac damage staging at follow-up; Table S1: Intra- and inter-observer variability for LVGLS assessment; Table S2: Follow-up echocardiographic characteristics according to cardiac damage staging.
