# Supplementary material for: Changes in Global Longitudinal Strain after TAVI: Additional Prognostic Value over Cardiac Damage in Patients with Severe Aortic Stenosis
Source: J Clin Med. 2024 Jul 5;13(13):3945. doi: 10.3390/jcm13133945 (PMC11242647; doi:10.3390/jcm13133945)
Supplement: Supplementary file 1 [file jcm-13-03945-s001.zip › jcm-3062120-supplementary.pdf]

Supplementary Figures.

Supplementary Figure S1. Incremental prognostic value of follow-up LVGLS over conventional cardiac damage staging at follow-up.

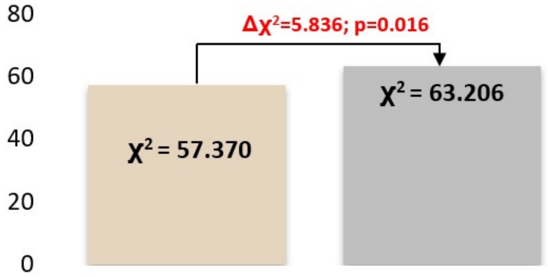

	Multivariate Analysis* Baseline model		Multivariate Analysis* Model 1	
	HR (95% CI)	p-value	HR (95% CI)	p-value
**Staging at follow-up**	1.245 (1.050-1.475)	**0.011**	1.201 (1.010-1.429)	**0.039**
**LVGLS at follow-up, %**	-	-	0.958 (0.925-0.992)	**0.015**

The figure shows the incremental prognostic value improvement of LVGLS at 6 months after TAVI over cardiac damage at 6 months after TAVI.

CI, confidence interval; HR, hazard ratio; LVGLS, left ventricular global longitudinal strain.

Bold values represent significant *p*-values (<0.05).

*Adjusted for male gender, coronary artery disease, use of diuretics, previous cardiac surgery, peripheral artery disease, smoking, mean aortic valve velocity, hemoglobin (g/dL), creatinine (mg/dL).

Supplementary tables.

Supplementary Table S1. Intra- and inter-observer variability for LVGLS assessment.

	Intra-class Correlation Coefficient (95% CI)
**Intra-observer Variability (N=15)**	
LVGLS	0.962 (0.911 to 0.986)
**Inter-observer Variability (N=15)**	
LVGLS	0.895 (0.773 to 0.960)

CI = confidence interval; LVGLS = left ventricular global longitudinal strain.

The intra-class correlation coefficients for intra- and inter-observer variability for LVGLS on 15 randomly selected patients were 0.962 and 0.895, demonstrating excellent agreement.

Supplementary Table S2: Follow-up echocardiographic characteristics according to cardiac damage staging

	**Total population (n=620)**	**Follow-up**					**p-Value**
		**Stage 0 (n=21)**	**Stage 1 (n=130)**	**Stage 2 (n=293)**	**Stage 3 (n=126)**	**Stage 4 (n=50)**	
**Heart rate (beats per minute)**	70.3 ± 12.8	69.0 ± 12.8	70.7 ± 12.5	68.9 ± 12.6	71.7 ± 12.7	74.6 ± 13.6	**<0.001**
**LV end-diastolic diameter, indexed (mm/m²)**	26.2 ± 3.9	22.7 ± 2.2§ǁ	25.3 ± 2.9	26.7 ± 3.9†	26.3 ± 4.0†	27.1 ± 5.3†‡§ǁ	**<0.0001**
**LV end-systolic diameter, indexed (mm/m²)**	17.9 ± 4.2	14.6 ± 2.1ǁ	17.1 ± 3.4§ǁ	18.2 ± 4.2‡	17.7 ± 4.3†‡ǁ	20.0 ± 5.6†‡§ǁ	**<0.0001**
**Septal wall thickness (mm)**	13.1 ± 2.2	13.0 ±1.6	13.4 ± 2.1	13.1 ± 2.0	13.2 ± 2.3	12.9 ± 2.9	0.415
**Posterior wall thickness, mm**	10.4 ± 1.8	9.8 ± 1.2	10.6 ± 1.6	10.5 ± 1.8	10.3 ± 1.8	10.3 ± 2.4	0.280
**Relative wall thickness (mm)**	0.4 ± 0.1	0.5 ± 0.1	0.5 ± 0.1	0.4 ± 0.1	0.4 ± 0.1	0.4 ± 0.1†‡§ǁ	**<0.001**
**LV end-diastolic volume (ml/m²)**	45.9 (38.5-56.4)	39.8 (36.5-44.8)	45.8 (37.3-53.2)	47.3 (38.8-59.4)	45.8 (38.2-53.6)	47.6 (36.5-62.6) ‡§ǁ	**0.001**
**LV end-systolic volume (ml/m²)**	17.5 (12.0-24.2)	11.9 (9.7-15.2)	16.8 (11.2-22.1) ǁ	18.5 (12.6-25.9)	17.1 (12.3-22.0) ‡	21.7 (14.0-33.7) †‡§ǁ	**<0.0001**
**LV mass index (g/m²)**	117.9 ± 29.8	88.9 ± 11.5	116.2 ± 26.8	120.3 ± 29.3	117.3 ± 30.2†	123.1 ± 37.1†‡§ǁ	**<0.0001**
**LV ejection fraction, %**	61.5 (53.3-69.3)	72.2 (60.0-75.5)	64.4 (54.8-70.3)	60.5 (54.0-68.7)	62.0 (52.2-70.3) †‡§	52.9 (43.8-62.0) †‡§ǁ	**<0.0001**
**LV global longitudinal strain, %**	16.3 ± 4.6	19.1 ± 3.9	17.5 ± 4.1	16.3 ± 4.5†‡	16.0 ± 4.9†‡§	12.8 ± 4.1†‡§	**<0.0001**
**E/e′ ratio**	18.8 (14.2-27.0)	11.0 (9.2-11.7)	16.5 (13.3-23.7)	19.8 (14.7-26.5)	22.7 (15.8-32.2)	17.5 (12.4-28.3) ‡	**0.006**
**Left atrial volume index (ml/m²)**	43.3 ± 16.8	24.0 ± 6.8	28.0 ± 4.5	48.3 ± 12.5†‡	51.0 ± 20.8†‡	44.7 ± 19.3†‡	**<0.0001**
**Significant mitral regurgitation, n (%)**	80 (13)	-	-	40 (14)	35 (28)	5 (11)	**<0.0001**
**Systolic pulmonary arterial pressure (mmHg)**	31.0 ±13.6	23.5 ± 8.9	23.4 ± 9.5	28.9 ± 11.4	43.2 ± 13.7	35.3 ± 14.5	**<0.0001**
**Significant tricuspid regurgitation, n (%)**	140 (23)	-	-	-	123 (98)	17 (36)	**<0.0001**

**Tricuspid annular plane systolic excursion (mm)**	19.9 ± 3.1	20.3 ± 2.5	20.1 ± 2.3	20.6 ± 2.6‖	20.4 ± 2.6§	13.7 ± 1.7†‡§‖	**<0.0001**
**Stroke volume index, ml/m^2**	43.6 ±13.8	44.7 ± 13.1	42.9 ± 12.2	45.4 ± 14.6‖	42.7 ± 13.8§	37.3 ± 11.5‡§‖	**<0.001**
**Mean aortic valve gradient (mmHg)**	9.8 ± 4.2	10.8 ± 5.0	10.2 ± 4.5	9.6 ± 4.0	10.1 ± 4.7	8.1 ± 3.1‡§	**<0.001**
**Peak aortic jet velocity (m/s)**	2.1 ± 0.4	2.2 ± 0.5	2.1 ± 0.5	2.1 ± 0.4	2.1 ± 0.4	1.9 ± 0.4‡§	**<0.001**

Continuous variables are presented as mean ± SD or median [interquartile range]. Categorical variables are expressed as numbers (%). The boldface values indicate statistical significance. LV, left ventricular.

**p*-Values depict differences between stages of cardiac damage, and are calculated by ANOVA and Kruskal–Wallis H test for continuous data (with normal and non-normal distributions, respectively), and by chi-square test for categorical data.

†*p*-Value <0.05 vs stage 0 with Bonferonni post hoc analysis. ‡ *p*-Value <0.05 vs stage 1 with Bonferonni post hoc analysis. §*p*-Value <0.05 vs stage 2 with Bonferonni post hoc analysis. ‖ *p*-Value <0.05 vs stage 3 with Bonferonni post hoc analysis.
